# Simultaneous Targeting Tumor Cells and Cancer-Associated Fibroblasts with a Paclitaxel–Hyaluronan Bioconjugate: In Vitro Evaluation in Non-Melanoma Skin Cancer

**DOI:** 10.3390/biomedicines9060597

**Published:** 2021-05-24

**Authors:** Barbara Bellei, Silvia Caputo, Emilia Migliano, Gianluca Lopez, Valeria Marcaccini, Carlo Cota, Mauro Picardo

**Affiliations:** 1 Laboratory of Cutaneous Physiopathology and Integrated Center of Metabolomics Research, San Gallicano Dermatologic Institute, IRCCS, 00144 Rome, Italy; silvia.caputo@ifo.gov.it (S.C.); gianluca.lopez@ifo.gov.it (G.L.); valeria.marcaccini@ifo.gov.it (V.M.); mauro.picardo@ifo.gov.it (M.P.); 2 Department of Plastic and Reconstructive Surgery, San Gallicano Dermatological Institute, IRCCS, 00144 Rome, Italy; emilia.migliano@ifo.gov.it; 3 Department of Dermatology, San Gallicano Dermatological Institute, IRCCS, 00144 Rome, Italy; carlo.cota@ifo.gov.it

**Keywords:** CAFs, skin cancer, tumor stroma, inflammation, microenvironment

## Abstract

Background: Cancer-associated fibroblasts (CAFs) facilitate many aspects of cancer development by providing a structural framework rich in bioactive compounds. There are emerging studies proposing a combination of conventional anti-cancer therapies directed against neoplastic cells to molecules targeting tumor microenvironments. Methods: The study evaluated the pharmacological properties of the anti-tumor agent paclitaxel conjugated to hyaluronic acid (HA) regarding non-melanoma skin cancer (NMSC) and the surrounding fibroblasts. This molecule, named Oncofid-P20 (Onco-P20), preferentially targets cells expressing high levels of CD44, the natural ligand of HA. Results: Consistent with paclitaxel’s mechanism of action involving interference with the breakdown of microtubules during cell division, highly sensitive carcinoma cells rapidly underwent apoptotic cell death. Interestingly, less sensitive cells, such as dermal fibroblasts, resisted the Onco-P20 treatment and experienced a prolonged growth arrest characterized by morphological change and significant modification of the gene expression profile. Onco-P20-treated fibroblasts exhibited reduced growth factor production, downmodulation of the Wnt signaling pathway, and the acquisition of a marked pro-inflammatory profile. Independently of direct exposure to taxol, in the presence of Onco-P20-treated fibroblasts or in their conditioned medium, carcinoma cells had a reduced proliferation rate. Similar to NHF, fibroblasts isolated from skin cancer lesions or from adjacent tissue acquired anti-neoplastic activity under Onco-P20 treatment. Conclusion: Collectively, our data demonstrate that Onco-P20, exerting both a direct and an NHF-mediated indirect effect on carcinoma cells, is a candidate for an innovative therapy alternative to surgery for the treatment of NMSC.

## 1. Introduction

Keratinocyte-derived non-melanoma skin cancer (NMSC), predominantly basal and squamous cell carcinoma, represents the most frequent malignant tumor worldwide [1]. Although it rarely metastasizes, its locally aggressive invasion frequently causes disfigurement, morbidity, and lowers the patient’s quality of life. In this selection of patients, pharmacological therapies are preferable to surgical treatment, radiotherapy, and other destructive techniques [2]. Medical treatments include topical imiquimod, 5-fluorouracil, and ingenol metabutate. Recently, smoothened Hedgehog pathway inhibitors have been approved for oral treatment of locally advanced or metastatic basal cell carcinoma [3]. New findings indicate the importance of the tumor microenvironment for the biological properties of the tumor, such as aggressive growth and local recurrence encouraging innovative therapeutic approaches. During malignant transformation, cancer cells acquire invasive properties, breach the basement membrane, and invade the underlying dermis. Consequently, dermal stroma and epidermal cancer cells establish unusual heterotypic cell–cell contacts and a dynamic cross-talk that leads to alterations of the host tissue [4,5]. Parenchymal injury associated with a nascent and growing tumor constantly activates stromal cells, driving the acquisition of a cancer-associated phenotype that neither spontaneously reverts to a normal phenotype nor undergoes cell death [6]. A particular feature of cancer stroma is the increased number of mesenchymal or fibroblastic cell types, pathologically activated and referred to as cancer-associated fibroblasts (CAFs). CAFs are embedded within an extracellular matrix (ECM), which is profoundly remodeled compared to the physiological one and is rich in immune cell infiltrates and blood/lymphatic vessels. The ECM not only provides structural support for the cells, but also participates in biochemical signal transduction through the binding of angiogenic and growth factors, whose availability also depends on ECM remodeling [7,8,9]. CAFs are characterized by a distinct activated phenotype and by expression of a variety of markers, such as fibroblast-activated protein-α (FAP-α), platelet-derived growth factor receptor-α and β (PDGFR-α and β), α-smooth muscle actin (α-SMA), and fibroblast-specific protein-1 (FSP1) [10,11,12]. CAFs in cancer tissues are morphologically similar to myofibroblasts, which are large spindle-shaped cells, activated during the wound healing process and found in sites of chronic inflammation [13,14]. Based on the similarity between tumor initiation and wound inflammation linked to myofibroblast activation, tumors have been defined as “wounds that never heal” [15]. This notion implies the reversibility of fibroblast activation and the possibility to target CAFs for cancer therapy [16]. Interestingly, fibroblasts both within or directly in contact with a tumor, as well as those from cancer-free tissue adjacent to tumors, exhibit a CAF phenotype [17]. This phenomenon could be explained by the intense release of bioactive molecules by tumor tissue and the consequent bi-directional dynamic cross-talk among cancer cells and surrounding cells. It has been suggested that the high secretory phenotype of CAFs play a key role in tumor progression, but increasing data also argue for their antitumor functions [18,19]. In a mouse model, reduced stromal content accelerates tumor growth and angiogenesis, and full depletion of CAFs induces immunosuppression [20,21,22]. The capacity to sustain cancer depends largely on an altered secretome, augmented pro-mitogenic peptide, regulation of inflammation, and ECM, whereas the capacity to inhibit cancer is expected to predominantly depend on the interaction with the immune system. An increased production of a vast repertoire of growth factors has been reported, with some overlapping with distinct tumor types including NMSC [6,22,23,24,25]. Among these, VEGF, PDGFs, EGF, FGFs, and Wnts drive tumor growth and vasculature [26]. CAFs also secrete cytokines and chemokines that recruit and modulate the function of immune cells in the tumor microenvironment [9,27]. The peculiarity of the inflammation associated with tumorigenesis processes demonstrates chronic, non-resolving characteristics [27]. However, similar to mesenchymal cells, inflammatory cells operate in conflicting modalities, being both tumor antagonizing and tumor promoting [28]. The balance between the pro-inflammatory and anti-inflammatory response is deeply implicated in the patient’s prognosis [29,30]. It is not fully clear if the dual nature of the cancer microenvironment reflects the contemporary presence of heterogenic populations or if differences reside in disease evolution. Thus, since CAFs co-evolve with genetically altered tumorigenic cells, it is possible that an early anti-tumor phenotype is replaced by a pro-tumorigenic one during disease progression. In line with the idea that CAFs co-evolve with tumor cells, it has been demonstrated that normal dermal fibroblasts could be “educated” to learn the CAF gene signature by tumorigenic cells both in vivo and in vitro [31]. On the other hand, in the skin, chronic and long-term exposure to UV radiation, a major environmental risk factor for skin cancer [32], occurs in the whole tissue, impacting pre-carcinogenic and resident stromal cells at the same time. Thus, in contrast to internal organs, skin fibroblasts undergo a continuous extrinsic stimulation. Therefore, in the skin, alterations in the stroma can precede (or act independently of) epithelial cell alterations, functioning as a driver of the tumorigenic process. Thus, activated fibroblasts may play a relevant role in both initiating and progressing skin carcinogenesis. Consequently, in order to control cancer, we need to focus not only on malignant cancer cells, but also on the benign microenvironment. Moreover, therapies against stromal targets may also be useful as an adjuvant strategy for patients receiving conventional surgery. Until now, most of the anti-stromal therapies have been designed to target neo-angiogenesis and immune response [33]. Currently, CAF-targeting strategies under clinical consideration are restricted to FAP-blocking molecules [34]. In this study, we investigated the effect of the anti-tumor agent paclitaxel (PTX) conjugated to hyaluronic acid (HA), the CD44 ligand, and a molecule named Oncofid-P20 (Onco-P20) on skin carcinoma cells, dermal fibroblasts, and CAFs. PTX, a microtubule-targeting drug, enhances the polymerization of tubulin and also interacts directly with microtubules, stabilizing them against depolymerization and enabling cells to form a normal mitotic apparatus [33]. Penetration-enhancing strategies, including liposomes, nanoparticles, implants, and albumin-bound PTX, have been proposed to bypass the poor water solubility of taxols. Improved skin penetration is desirable due to the proved efficacy of PTX against melanoma and non-melanoma skin cancer [35,36,37,38]. HA-conjugate is a highly biocompatible strategy to increase the bioavailability and pharmacokinetics of active molecules, especially in tissues presenting a high hyaluronan receptor density, such as the skin. Moreover, in many cancers of epithelial origin, the up-regulation of CD44 has been observed [39]. In addition, CD44 is abundantly expressed by CAFs, particularly in the hypoxic context [40]. It has been previously demonstrated that dermal fibroblasts primed with PTX are capable of releasing functionally active PTX, reducing melanoma cells [41,42]. In line with this set of preliminary data, using Onco-P20, we analyzed the impact of PTX treatment in the context of NMSC. We demonstrated that Onco-P20 combines, in a single molecule, two synergic activities: direct anti-tumor efficacy on keratinocyte-derived skin cancer and an indirect anti-tumor activity dependent on the modulation of CAFs’ secretory profile. Based on the HA-CD44 interaction, Onco-P20 targets fibroblasts more efficiently than free PTX, representing a valid alternative to surgery for the treatment of non-melanoma skin carcinoma.

## 2. Materials and Methods

### 2.1. Ethic Statement

The Declaration of Helsinki Principles were followed, and patients gave written informed consent to collect samples of human material for research. Furthermore, the Institutional Research Ethics Committee (Istituti Regina Elena e San Gallicano) approved all research activities involving human subjects.

### 2.2. Preparation of Hyaluronan–Paclitaxel Bioconjugate

The preparation of a HA–paclitaxel bioconjugate (Oncofid-P20) with ~20% *w*/*w* of paclitaxel (taxol) loading has been previously described [38]. In brief, Oncofid-P20 (Onco-P20) is a chemical conjugate between hyaluronic acid (Mw ranging between 100 and 220 kDa) and paclitaxel, covalently bound by an ester linkage through a molecular spacer. Onco-P20 and paclitaxel (donated by Fidia Farmaceutici, Abano Terme, Italy) were diluted with DMSO. Solutions were further diluted at each experimental day in order to achieve a 0.05% final DMSO concentration. Hyaluronic acid (HA) used as reference to compare receptor stimulation to Onco-P20 treatment had a molecular weight of 403 kDa (BioWORLD Inc., Dublin, HO, USA). An equivalent amount of hyaluronan was calculated considering ~80% *w*/*w*.

### 2.3. Cell Cultures

Primary cultures of normal human keratinocytes (NHK) and fibroblasts (NHF) were isolated from human skin fragments obtained from surgery. Briefly, skin was catted into approximately 4 mm^2^ sized pieces and digested overnight at 4 °C with dispase (2.5 mg/mL) to separate the epidermis from the dermis. Then, keratinocytes were dissociated from the epidermis by trypsin and propagated in serum-free M154 medium with Human Keratinocytes Growth Supplement (HKGS) supplements (Cascade Biologics Inc., Portland, OR USA), Ca^2+^ (0.07 mM), and antibiotics. The dermis was digested with collagenase for 2 h at 37 °C, and the obtained NHF were maintained in a culture with DMEM (EuroClone S.p.A., Milan Italy), supplemented with 10% FBS and antibiotics (Hyclone Laboratories, South Logan, UT, USA). Cancer-associated fibroblasts (perilesional CAFs) and paired normal associated fibroblasts (NAFs) were isolated from 6 tumor samples of patients, 7 SCC (SCC63-TG; SCC376-DMG; SCC439-PG; SCC1300-UC; SCC233-FG; SCC138-CI; and SCC316-BA), and 2 BBC (BCC263-DP and BCC233-FG) using the same method. After having surgically separated peritumoral tissue from the tumor lesion, the above described 6 carcinoma samples were washed three times with PBS, then dissected into approximately 1–2 mm^2^ sized pieces and digested using 5 mL of a 0.1% trypsin solution (Gibco, CA, USA). After variable digestion time (2–6 h), the homogenate was collected and passed through a 70 µm strainer and cultured in M154 plus HKGS. A small portion of the carcinoma (~10%) was digested with collagenase to obtain intra-tumoral fibroblasts (lesional CAFs). A431 and SCC1300-UC cell lines were cultured in DMEM containing 10% FBS or in M154 plus supplements where indicated. Images were recorded using an Axiovert 25 inverted microscope (Carl Zeiss, Oberkochen, Germany) and a Power Shot G5 digital camera (Canon, Inc., Tokyo, Japan).

### 2.4. Proliferation Assays

Briefly, 2.5 × 10^4^ keratinocytes or 0.8 × 10^4^ fibroblasts were seeded into the 24-well plates for 24 h to adhere. Then, growth medium was changed with fresh medium containing treatments (or not for control cells) at the appropriate concentrations. The culture medium and drugs were refreshed twice a week. At the experimental end point, cells were incubated with 3-(4,5 dimethylthiazol)-2,5-diphenyl tetrazolium bromide (MTT) for 2 h. After this time, the medium was removed, and the resulting crystals were solubilized in DMSO. The absorbance was measured at 570 nm with a reference wavelength of 690 nm. Absorbance readings were subtracted from the value of blank wells, and results were calculated as a percentage of absorbance in respect to control samples. Experiments were performed in duplicates.

### 2.5. Coomassie Staining

Cell monolayers were fixed with 4% paraformaldehyde for 20 min at room temperature, followed by an incubation period of 30 min at room temperature with Comassie Brillant Blue Staining Solution (BioRad Laboratories Inc. Milan, Italy). Then, for the de-staining step, cells were rinsed with PBS for 1 h with gentle agitation. Images were recorded using an Axiovert 25 inverted microscope (Carl Zeiss, Oberkochen, Germany) and a Power Shot G5 digital camera (Canon, Inc. Amstelveen, The Netherlands).

### 2.6. Preparation of CAF Conditioned Medium (CM)

CAFs were plated into a 10 cm^2^ dish, and a conventional culture was carried out for 24 h before Onco-P20 treatment. Subsequently, fresh DMEM + 10% FBS and treatment (or not for control cells) was replaced twice a week. After 2 weeks and an additional 48 h period without treatment, the medium was replaced with DMEM without FBS before CM harvesting. The supernatant was collected, centrifuged at 1000 rpm, and filtered with a 0.22-μm membrane for sterilization. CM of untreated proliferating fibroblasts was used as a control medium. Experiments were performed in duplicates.

### 2.7. Trans-Well Co-Culture

Briefly, 12 mm of trans-well, with a 3.0 μm pore membrane insert, was used with CAFs and cancers cells alternatively in the lower or upper compartment. A total of 2.0 × 10^4^ fibroblasts per well were seeded in the lower compartment, or alternatively, 0.8 × 10^4^ fibroblasts were seeded in upper compartment and incubated at 37 °C with 5% CO2 overnight. Then, fresh DMEM + 10% FBS and treatment (or not) was replaced twice a week. After 2 weeks, DMEM was replaced with M154 without treatment. After 48 h, trans-well inserts containing 2.0 × 10^4^ carcinoma cells were added. Otherwise, 2.0 × 10^4^ carcinoma cells were seeded in the lower compartment before insert addition. After 96 h, an MTT assay was developed. Experiments were performed in duplicates.

### 2.8. Western Blot Analysis

Cell extracts were prepared with an RIPA buffer containing proteases and phosphatases inhibitors. Proteins were separated on SDS-polyacrylamide gels, transferred to nitrocellulose membranes, and then treated with the following primary antibodies: mouse monoclonal CD44, anti-β-catenin, anti-cyclinE (1:1000) (Santa Cruz Biotechnology Inc., Santa Cruz, CA, USA), anti-bFGF (1:500) (Upstate Biotechnology, Inc., Lake Placid, NY, USA), anti-p53, anti-cyclinD1 (1:1000) (Dako, Agilent Technology Italia, Milan, Italy), rabbit polyclonal anti-p21, anti-p27 (1:500), anti-cyclinB1 (1:1000) (Cell Signaling Biotechnology), and goat anti-KGF (1:200), (Santa Cruz Biotechnology) antibodies. An anti-β-actin mouse monoclonal antibody (1:5000) (Sigma Aldrich, Merck KGaA, Darmstadt, Germany) was used to normalize the protein content. Horseradish peroxide-conjugated goat anti-mouse, goat anti-rabbit, or bovine anti-goat secondary antibody complexes were detected by chemiluminescence (Cell Signalling Technology, Beverly, MA, USA). Imaging and densitometric analysis were performed with a UVITEC Mini HD9 acquisition system (Alliance UVItec Ldt, Cambridge, UK).

### 2.9. Elisa Assay

The growth factors and bioactive molecules released in the culture medium were measured after two weeks of treatment using commercially available enzyme-linked immunosorbent assay (ELISA) kits, according to the manufacturer’s instructions: HGF (Cusabio Technology LLC, Baltimore, MD, USA), IGFBP4 and 6 (Aviva Systems, Biology, San Diego, CA, USA), and VEGF-A (eBioscience, Inc., San Diego, CA, USA). Proliferating fibroblasts were used as the control. Cells were incubated for 48 h with DMEM without FBS before medium collection. Results were normalized against the protein concentration.

### 2.10. Immunofluorescence Analysis

Cells on coverslips were fixed with 4% paraformaldehyde for 20 min at room temperature, followed by 0.1% Triton X-100 to allow cell permeabilization. Cells were then incubated with anti-PML rabbit polyclonal (1:500) (Santa Cruz Biotechnology), for 1 h. Primary antibodies were visualized using anti-rabbit IgG Alexa Fluor 488 (BD Bioscences, Milan, Italy). Incubation with a secondary antibody alone was used as a negative control. Nuclei were visualized with 4′,6′-diamino-2-phenylindole (DAPI). Fluorescence signals were recorded using a CCD camera (Zeiss, Oberkochen, Germany).

### 2.11. Semi-Quantitative RT-PCR and Gene Expression Array Cards Analysis

Total RNA was extracted using Aurum Total mini kit (BioRad, Milan Italy). cDNA was synthesized from 1 μg of total RNA using the FirstAid kit (Fermentas, ThermoFisher Scientific, Waltham, MA, USA) and loaded on 384-well microfluidic cards designed to perform probe-based TaqMan real-time PCR on Applied Biosystems^®^ QuantStudio^TM^ 7 Flex instruments (Thermo Fisher Scientific, Roma, Italy). Cards were configured with selected primers and probe sets to analyze 93 target genes and three housekeeping genes (18s rrna, gliceraldeide-3-fosfato deidrogenasi (GAPDH), and β-actin). Results were analyzed using a cloud-based platform one-way ANOVA statistical test with thresholds of >2.0 and <0.5 fold-change and *p* ≤ 0.05. For semi-quantitative real-time PCR, cDNA was amplified using SsoAdvanced Universal Syber Green Supermix (BioRad), containing 25 pmol of forward and reverse primers using a CXF96 Touch Cycler (BioRad). All samples were tested in triplicate. Amplification of the β-actin transcript from each sample was included as an internal control. Sequences of primers (intron spanning) can be found in Table S1. For each gene, the assessment of quality was performed by examining the end-point PCR melt curves to ensure product specificity.

### 2.12. Flow Cytometry Analysis

Cell death and apoptosis were analyzed by the annexin-V FITC/propidium iodide (PI) double-staining method after 48 h of treatment. Cells were harvested by trypsinization, suspended in the staining buffer (10 mm HEPES/NaOH, pH 7.4, 140 mm NaCl, 2.5 mm CaCl_2_), stained with FITC-labeled annexin V and PI for 15 min at RT in the dark, and then kept on ice until being analyzed. For cell cycle distribution, after cell harvesting by trypsinization, cells were fixed in cold 70% ethanol for 10 min and then stained with propidium iodide (PI) solution (1 μg/μL PI and 0.125% RNaseA; Sigma Aldrich, St. Louis, MO, U.S.A.) at room temperature for 15 min. A total of 20,000/sample cells were analyzed using a FACS Calibur instrument (BD) equipped with a 488 nm argon ion laser. The percentage of cells in each phase of the cell cycle was determined using FlowJo software v8.0. The amount of membrane-bound CD44 was measured by incubating unfixed cells with a CD44-PE antibody (BD Biosciences) before the cell wash and cytofluorimetric analysis. Unstained cells were used as a negative control.

### 2.13. Cytokines Protein Array

The expression of 20 human cytokines was analyzed using a commercially available antibody array system (RayBio^®^ C-Series Human Inflammation Array C1 Map, RayBiotech Life, Inc., GA, U.S.A.) that uses membrane-bound cytokine-specific antibodies to capture cytokines in biological fluids. The procedure was performed according to manufacturer’s instructions. Cells were seeded in 10 cm culture dishes and treated (or not) with Onco-P20 0.15 μg/mL for 2 weeks. After removing drug, cells (and untreated proliferating control fibroblasts), cells were maintained in serum-free medium for 48 h. The cytokine array membranes were blocked in 2 mL 1 × blocking buffer for 30 min at room temperature (RT), and then they were incubated with 1 mL of the conditioned medium at 4 °C overnight. The medium was then decanted from each container, and the membranes were washed three times with 2 mL 1 × wash buffer I, followed by two washes with a 2 mL 1 × wash buffer II at room temperature. Following this, the membranes were incubated in biotin-conjugated primary antibodies for 2 h at RT and then washed as described above before incubation in 1:1000-diluted horseradish peroxidase-conjugated streptavidin for 2 h. The membranes were then washed thoroughly and incubated with a chemiluminescent ECL substrate at RT for 5 min. Imaging and densitometric analysis were performed with a UVITEC Mini HD9 acquisition system (Alliance UVItec Ldt, Cambridge, UK).

### 2.14. Statistical Analysis

Results in the figures are representative of several experiments we performed with at least five cell lines from different donors. Quantitative data were reported as mean ± standard deviation (SD). A student *t*-test was used to assess statistical significance, with thresholds of * *p* ≤ 0.05 and ** *p* ≤ 0.01.

## 3. Results

### 3.1. Effect of Oncofid-P20 Treatment on Cell Proliferation and Viability

**Figure 1:**
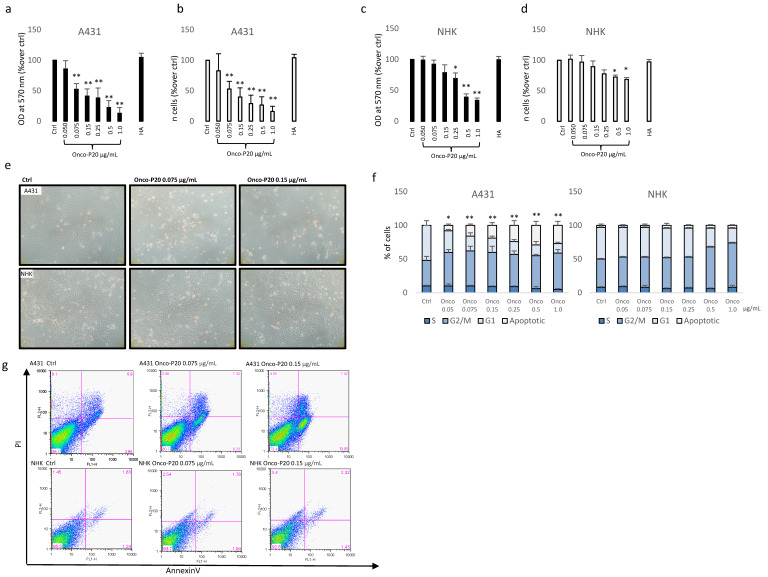
MTT assay (**a**) and cell count (**b**) of A431 cells treated (72 h) with increasing concentrations of Onco-P20 (range 0.05–1.0 μg/mL) or HA 1.0 μg/mL, equivalent to the HA concentration at the higher doses of Onco-P20. Data are reported as optical density (OD). Data represent the mean ± SD of three independent experiments performed in duplicate. MTT assay (**c**) and cell count (**d**) of NHKs treated (72 h) with increasing concentrations of Onco-P20. Histograms represent the mean ± SD of three NHK cell lines isolated from different donors. (**e**) Microscopic images of normal and carcinoma cells after 72 h of treatment with Onco-P20. (**f**) Cell cycle distribution of Onco-P20-treated normal and carcinoma cells. (**g**) Apoptosis was evaluated by FACS analysis using annexin V/iodide propidium staining. Dot plots show one representative experiment performed 24 h after Onco-P20 treatment. Statistical significance versus untreated control is reported as * *p* < 0.05 and ** *p* < 0.001. In the case of (**g**), statistical significance represents the difference of apoptotic cells in A431 versus NHKs.

A431 human squamous carcinoma cells, a commonly used model for studies of skin cancer, and normal keratinocytes (NHK) from healthy subjects were treated with a broad panel of an Onco-P20 concentration (0.05–1 µg/mL) for 72 h before an MTT assay and cell count. The MTT assay showed a strong reduction in NAD(P)H-dependent cellular mitochondrial activity in Onco-P20-treated carcinoma cell cultures that was correlated with a significant reduction in the number of cancer cells at all the concentrations tested (Figure 1a,b). By contrast, at the experimental end point, in NHK, Onco-P20 treatment mildly impacted metabolic function, and the number of cells was modestly reduced (Figure 1c,d). Hyaluronic acid alone did not exert any significant modification of cell viability or proliferation, indicating that this portion of the molecule was not implicated in the biological activity observed. Since, in addition to cell counts, the microscopic observation of cell cultures highlighted marked differences when comparing normal and tumor cells (Figure 1e), we additionally evaluated the cell cycle distribution, demonstrating G2/M accumulation in both cell types and extensive cell death only in cancer cells (Figure 1f). A significant increase in the number of apoptotic cells was confirmed by AnnexinV/PI staining, proving the selective cytotoxic effect on tumor cells (Figure 1g). Thus, while in A431 carcinoma cultures, metabolic activity measurements and the number of apoptotic cells were correlated, indicating that cell death was the main mechanism of Onco-P20’s function, in normal keratinocytes in all the concentrations tested, the treatment reduced metabolic activity without affecting cell viability. However, high doses of Onco-P20 (>0.25 µg/mL) impacted keratinocytes’ proliferation capability, as evidenced by cell accumulation in the G2/M cell cycle phases.

**Figure 2:**
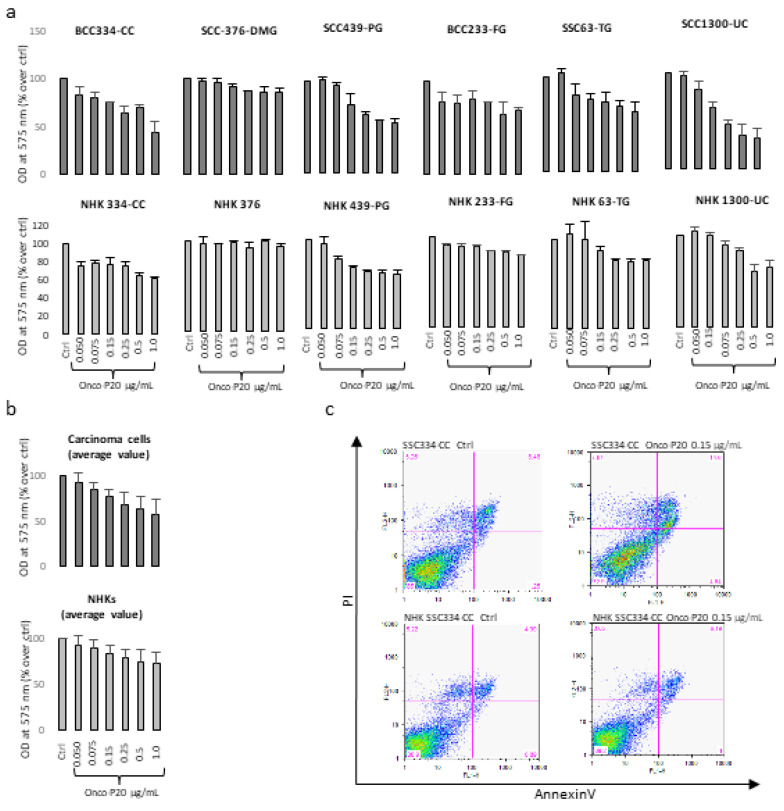
(**a**) MTT assay of several different NMSC cell lines and patient-matched NHKs after 72 h of treatment with Onco-P20 (range 0.05–1.0 μg/mL). Data are reported as optical density (OD). (**b**) Data are also reported as mean value ± SD. Experiments were performed in duplicate. (**c**) AnnexinV/iodide propidium staining evidenced apoptotic cell death only in carcinoma cells. Dot plots show one representative experiment performed 24 h after Onco-P20 treatment.

Since established cell lines maintained in culture for a long period frequently present evidence of deviations from the phenotype of the originating tumor, we used a collection of specimens from patients well-characterized for disease stage and followed up to isolate low-passage tumor cell lines and patient-matched normal keratinocyte cultures. In this case, the cytotoxic effect against short-term carcinoma cell cultures showed an overall lighter intensity compared to A431 cells and the MTT assay, which does not discriminate between cytotoxic and cytostatic effects, and which displayed a modest difference between normal and carcinoma cells (Figure 2a,b). By contrast, AnnexinV/PI staining confirmed that Onco-P20 preferentially reduced the viability of neoplastic cells (Figure 2c). Since A431 cell cultures and fresh isolated carcinoma cells were cultured in non-overlapping growth conditions (DMEM supplemented with FBS and chemically defined M154 medium supplemented with HKGS, respectively; see Section 2), we addressed the question of whether the observed differences could be attributed to the culture medium composition or to distinctive biological properties. In this case, we used A431 and SCC1300-UC, one of the low passage SCC cell lines isolated in our laboratory, capable of proliferating both in defined media (M154 plus HKGS) and in high calcium FBS-containing DMEM medium, to evaluate the impact of cell medium composition on Onco-P20 treatment.

**Figure 3:**
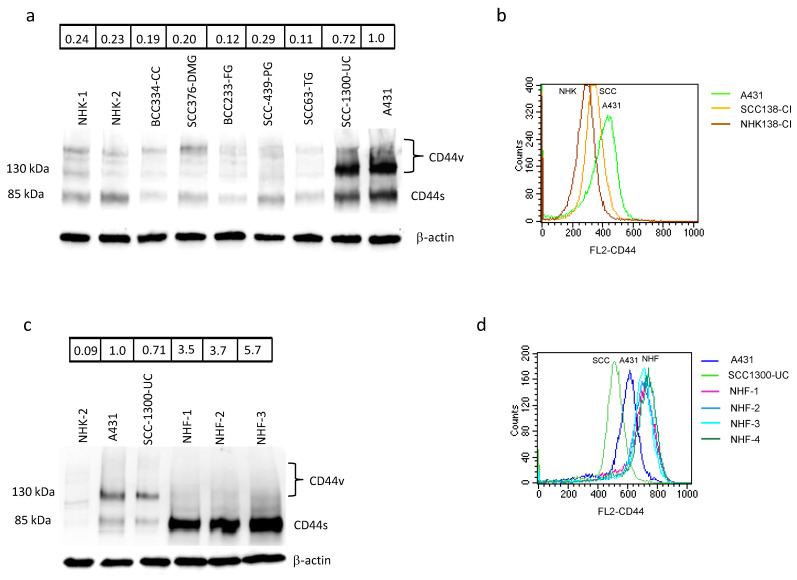
(**a**) Western blot and densitometric analysis of CD44 expression in NHKs and carcinoma cells. Data evidenced that A431 and SCC1300-UC patient-derived cells expressed the CD44v isoform in addition to the standard isoform (CD44s). (**b**) One representative immunostaining of plasma membrane CD44 expression in carcinoma cells and normal keratinocytes. Data demonstrated lower intensity in freshly isolated carcinoma cells (SCC138-CI) compared to the A431 carcinoma cell line. NHKs isolated from healthy skin of the same patient (NHK138-CI) presented the lowest quantity of CD44. (**c**) Western blot and densitometric analysis of CD44 expression in carcinoma cells and fibroblasts. Similar to (**a**), semi-quantitative analysis of CD44 staining showed high expression in all NHF cell cultures analyzed, compared to A431, and in freshly isolated carcinoma cells (SCC1300-UC). (**d**) One representative immunostaining of plasma membrane CD44 expression in carcinoma cells and normal fibroblasts.

As shown in Supplementary Figure S1, the sensitivity to Onco-P20 treatment was attenuated in the M154-defined medium compared to DMEM plus serum (Figure S1a) and inversely correlated with the proliferation rate (Figure S1b). In fact, cell culture composition influenced cell growth, demonstrating a significantly slower growth kinetic in the chemically defined medium, which correlated with lower pharmacological activity. However, responsiveness to Onco-P20 is also an intrinsic characteristic of different cell lines related to the CD44 level of expression that is not necessarily over-expressed in skin carcinomas compared to normal cells [43]. In fact, Western blot analysis confirmed that most patient-derived carcinoma specimens presented a modest level of CD44 (Figure 3a). Interestingly, A431 and SCC1300-UC abundantly expressed the 130 kDa isoform (CD44v) in addition to the CD44 standard isoform (CD44s). Furthermore, FACS analysis of live cells performed without membrane permeabilization confirmed that the functionally active HA receptor was barely present on the NHK cell surface (Figure 3b). Accordingly, considering that the relative abundance of CD44 was not particularly elevated in the keratinocyte lineage, the hyaluronan conjugate formulation failed to demonstrate any significant gain of function in comparison to conventional free paclitaxel (Figure S2).

CD44-overexpressing cells comprised not only tumor cells and cancer stem cells, but also CAFs [44,45,46,47,48]. Strongly CD44-positive CAFs are particularly frequent in tumor hypoxic areas [40]. Since dermal fibroblasts also physiologically expressed a high level of this receptor (Figure 3c,d), we evaluated the effect of Onco-P20 on normal human fibroblasts (NHF).

**Figure 4:**
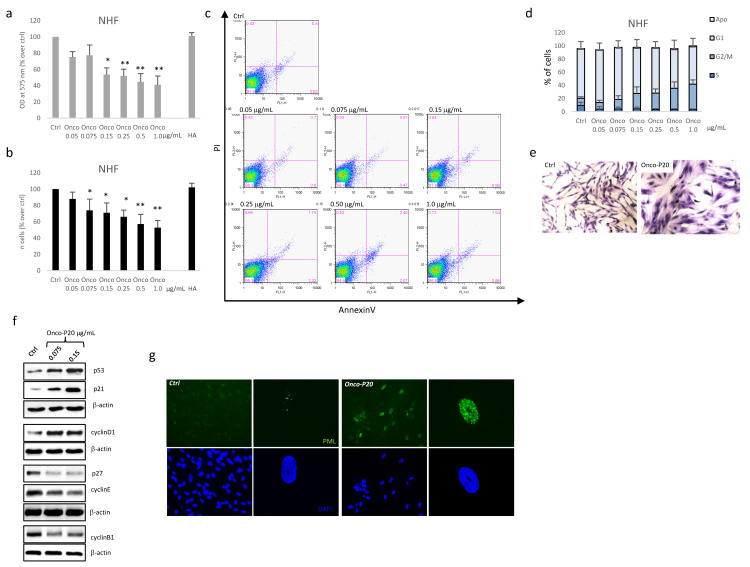
(**a**) MTT assay and cell count (**b**) of several different NHF cell lines treated (72 h) with increasing concentrations of Onco-P20 (range 0.05–1.0 μg/mL) or HA 1.0 μg/mL, equivalent to the HA concentration at the Onco-P20 higher doses. (**c**) FACS analysis using AnnexinV/iodide propidium staining excluded apoptosis. Dot plots show one representative experiment performed 24 h after Onco-P20 treatment. (**d**) Histograms show the mean ± SD of six independent experiments performed with cells isolated from six different donors. (**e**) Representative images of Coomassie staining of fibroblasts after Onco-P20 treatment for 14 days and of control active proliferating cells. Original magnification 20×. Statistical significance versus the untreated control is reported as * *p* < 0.05 and ** *p* < 0.001. (**f**) Western blot analysis of cell cycle progression regulators. (**g**) Immunofluorescence analysis of PML expression and of PML nuclear body organization. Nuclei were stained with DAPI. Original magnification of 20× and 63× for greater detail.

For this purpose, and in line with the patients’ characteristics, we used adult fibroblasts isolated from a photo-exposed area of geriatric donors. NHF treated with Onco-P20 (0.05–1.0 µg/mL) for 72 h displayed attenuation of mitochondrial activity and a significant reduction in cellular proliferation compared to control cells (Figure 4a,b). Similarly, to normal keratinocytes, fibroblasts did not show acute cytotoxicity as measured by AnnexinV/PI double staining (Figure 4c). Cell cycle analysis evidenced a marked accumulation in the G2/M phase (Figure 4d). In addition, evidence of cell hypertrophy (a large cell morphology) appeared at days 3–5 and persisted for the entire treatment period (Figure 4e). Increased expression of p53 and p21, as well as the presence of several promyelocytic leukemia protein (PML)-nuclear bodies, typical nuclear matrix structures implicated in the induction of the senescence process [47,48,49], confirmed the diminished proliferative propensity of the Onco-P20-treated cells (Figure 4f,g). In contrast to p21, another important cell cycle checkpoint CDK inhibitor, p27, was decreased. Cyclin E, which is required and rate liming for S phase entry, was slightly downregulated. We further analyzed the protein level of cyclins in the Oncofid-P20-treated cells. The level of cyclin D1, which promotes progression through G1/S phases, was elevated, while cyclin B1, promoting G2/M transition, was barely present [45,46]. In accordance with previous studies [50,51,52], the cyclin profile was consistent with G2 arrest.

**Figure 5:**
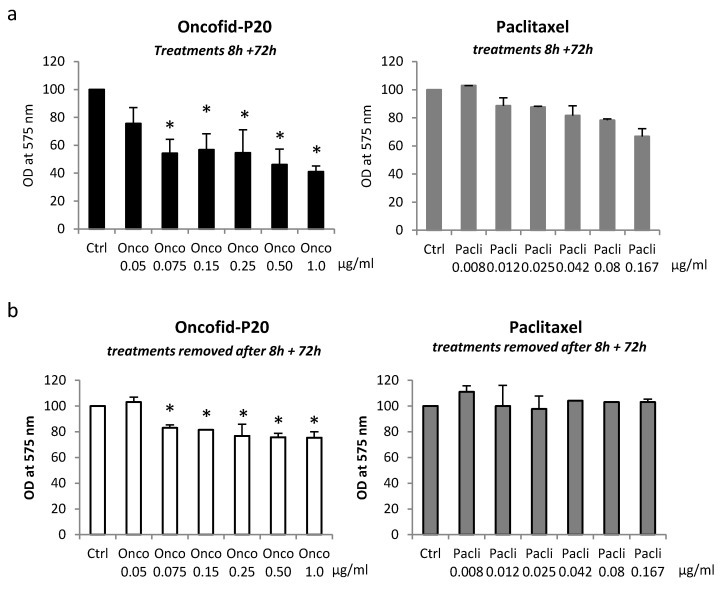
(**a**) MTT of NHFs treated for 8 h with Onco-P20 or PTX before the drug was washed out (**a**) or treated continuously for 72 h with identical treatments (**b**). Data represent the mean ± SD of three independent experiments performed in duplicate. Statistical significance expressing Onco-P20 versus PTX-treated cells is reported as * *p* < 0.05.

However, fibroblasts seem to be long-term arrested rather than showing irreversible senescence, since, after the removal of treatment, the cells resumed proliferation even in the case of prolonged exposure to Onco-P20 (Figure S3). Finally, Onco-P20 activity was compared with that of an equal amount of free paclitaxel. Following 72 h of drug exposure, unconjugated paclitaxel exhibited a lower effect on NHF (Figure 5a). Thus, due to high levels of CD44 expression on the membrane surface of mesenchymal cells, Onco-P20 exerted a stronger pharmacological activity than free paclitaxel. Moreover, to simulate drug availability in the case of topical application on patients’ skin, we exposed NHF to treatments (Onco-P20 or paclitaxel) for a defined short period (8 h) before placing cell cultures in a drug-free medium. In this case, using the same end point of previous experiments, we observed a reduced proliferation exclusively in Onco-P20-treated cells, whereas paclitaxel failed to modify fibroblast proliferation (Figure 5).

### 3.2. Onco-P20 Deeply Modifies Fibroblasts’ Genes and Protein Expression Profile

Given that fibroblasts could impact keratinocytes’ homeostasis through diffusible molecules, we assessed the impact of Onco-P20 on the expression of secreted proteins involved in tumor progression. Under these circumstances, several growth factors were differentially expressed in Onco-P20-treated fibroblasts versus control samples (Table 1).

**Figure 6:**
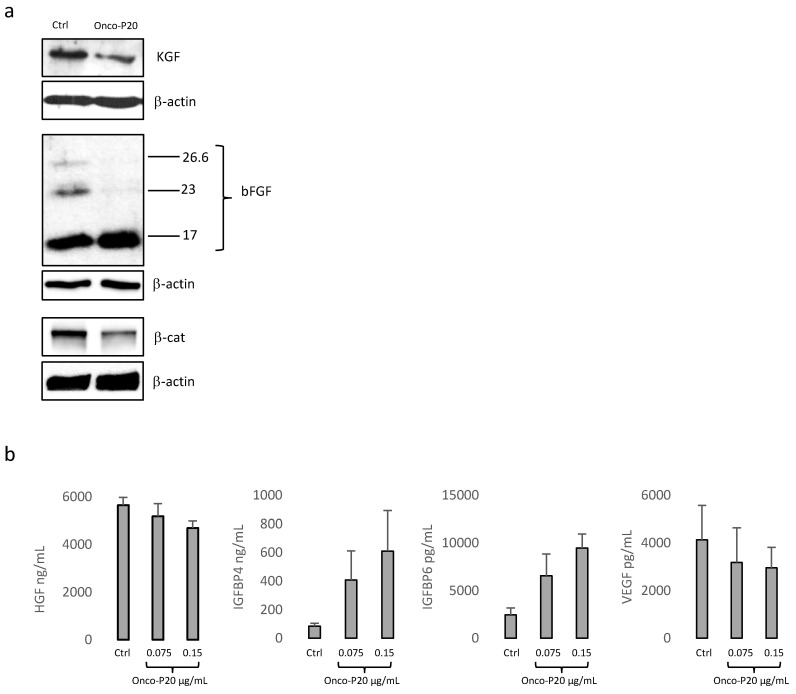
(**a**) One representative Western blot analysis demonstrating the reduction in growth factors and β-catenin expression in Onco-P20-treated (0.15 μg/mL) NHFs. Proteins were extracted after 2 weeks of treatment. (**b**) Immuno-enzymatic quantification of IGFBP4, IGFBP6, and VEGF released in the cell culture medium. After 2 weeks of treatment, cells were incubated for an additional 48 h with DMEM without FBS before medium collection. Results were normalized against the protein concentration.

Among these, HGF was strongly down-regulated both at the mRNA and protein levels (Figure 6a). Additionally, the high molecular weight of bFGF, frequently associated with a poor prognosis in various human cancers [53,54], was reduced by Onco-P20 (Figure 6b), whereas its mRNA was unmodified. However, since high molecular weight isoforms of bFGF are associated with highly proliferative phenotypes of fibroblasts [50], it is possible that the observed difference reflected an autocrine control of cell growth. Similarly, KGF was decreased mostly at the protein level. The EGF transcript was near the detection limit in most of the fibroblast cultures and slightly reduced by the treatment. IGF was unchanged, whereas members of the insulin-like growth factor binding protein (IGFBP) superfamily, a group of secreted proteins capable of binding IGFs and modulating the mitogenic, anti-apoptotic, and metabolic actions of IGFs, were found to be strongly increased. In particular, the IGFBP3, IGFBP4, IGFBP5, IGFBP6, and IGFBP7 mRNAs were significantly up-modulated. The immuno-enzymatic quantification of IGFBP4 and 6 corroborated the gene expression analysis (Figure 6a). The possible repression of neo-angiogenesis is supported by the reduced expression of vascular endothelial growth factors (VEGF) at the mRNA (Table 1) and protein levels (Figure 6b).

The Wnt/β-catenin signaling pathway, known to be activated in CAFs [55,56], was down-modulated in Onco-P20-treated fibroblasts, as demonstrated by decreased β-catenin, the pivotal molecule of the Wnt signaling pathway (Figure 6a).

Accordingly, the expression of some negative regulators of the pathway, such as Wnt5a, DKK1, and SFRP2 pathways, were increased, suggesting an autocrine mechanism of Wnt signaling regulation. By contrast, TGFβ expression was unchanged. Among the CAF markers, PDGFα and β were reduced, whereas the expression of FAP1 and αSMA was increased and unmodified, respectively. A strong increase in IL1α, IL1β, IL6, and IL8 suggests the activation of inflammatory pathways. Treatment with HA alone did not exert overlapping results, confirming that an Onco-P20-induced phenotype was attributable to taxol. By contrast, free paclitaxel induced a similar modification of the gene expression profile to that of Onco-P20 (data not shown). To further investigate signal transduction pathways involved in cancer–fibroblasts cross-talk, we investigated a wide range of cytokines and chemokines using gene expression array cards and an antibody membrane array. Among the 93 mRNAs studied, 38 were undetectable in most or all of the samples and were discarded from the analysis, 13 were unmodified by the treatment, 2 were slightly decreased, and 40 were enhanced (>1.5 fold-increase). Of these highly expressed transcripts, the differences for 17 reached statistical significance (*p* ≤ 0.05 or *p* ≤ 0.01) (Table 2).

Most of the up-regulated proteins, including KLK14 and members of the MAP kinase family, such as MAPK8 (JNK1) and MAPK14 (p38α), are implicated in the IL1 pathway. The expression of IL1R1 in the tumor microenvironment, the main receptor of IL1α and β, due to the interconnection with the NF-KB and MAP kinase pathways, plays contrasting roles in different tumor stages [57]. Sustained PTGS2 (cox-2) expression confirmed the pro-inflammatory profile of the NHF Onco-P20-induced phenotype. Annexin1A (ANXA1), increased by Onco-P20, has been proposed as a tumor suppressor in head and neck squamous cell carcinomas [58]. However, in prostatic cancer, ANXA1-exacerbated expression of this marker has been correlated with a high amount of cancer stem cells [59]. Interestingly, CD40, an antigen frequently lost by basal and squamous cell carcinomas during tumor escape from activated T cells, was augmented by Onco-P20 treatment. Intercellular adhesion molecule-1 (ICAM1, CD54), a receptor that supports leukocyte accumulation in inflamed tissue, turned out to be strongly enhanced, whereas vascular cell adhesion molecule-1 (VCAM), another mediator of leukocyte trafficking, was slightly increased. Additionally, high LTA4H that catalyzes the hydrolysis of epoxide LTA4 to LTB4, which mainly functions as a neutrophil, macrophage, and T lymphocyte chemoattractant, confirmed the inflammation-enhancing effect of Onco-P20. The antibody array used to quantify the secreted inflammatory factors confirmed a significant rise in IL6 and IL8 production, whereas IL1α and ILβ appeared moderately increased at the protein level. Additionally, the secretion of CCL11 (Eotaxin-1), a chemokine implicated in eosinophil recruitment [60], was up-regulated by Onco-P20 (Table 3, Figure S4). Lastly, CCL1, whose release by fibroblasts has been linked to bladder cancer cell invasion [61], was significantly diminished.

### 3.3. Secretome of Onco-P20-Treated Fibroblasts Modulates Carcinoma Cell Growth

**Figure 7:**
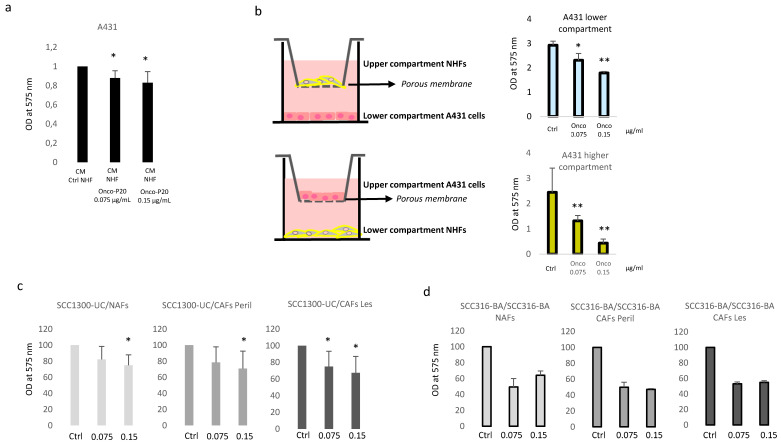
(**a**) MTT assay of A431 carcinoma cells grown in the presence of CM collected from Onco-P20-treated fibroblasts. After 2 weeks and an additional 48 h period without treatment, the medium was replaced with M154 without supplements before CM harvesting. CM was diluted 1:5 in fresh M154. CM of untreated fibroblasts was used as a control medium. Histograms represent the mean ± SD of three independent experiments performed with three different NHF cell lines at the experimental end point (72 h). (**b**) Schematic representation of the trans-well systems used for co-culture experiments. MTT assay after 72 h of A431 cells in the co-culture demonstrated dose-dependent anti-cancer activity of Onco-P20-treated normal fibroblasts. (**c**) MTT assay after 72 h of SCC1300-UC carcinoma cells in the co-culture with fibroblasts isolated from uninvolved (NAFs), perilesional, and lesional areas (CAFs) of carcinoma patients. Results report data relative to three independent experiments performed with three different donors. (**d**) MTT assay after 72 h of SCC316-BA carcinoma cells in the co-culture with matched fibroblasts isolated from uninvolved (NAFs), perilesional (CAFs Peril), and lesional (CAFs Les) areas of the same donor (SCC316-BA). Statistical significance versus untreated control cells is reported as * *p* < 0.05 and ** *p* < 0.001.

Cancer and stromal cells communicate mainly by a complex bidirectional cross-talk that evolves during disease progression and the diffusion of soluble factors through the basement membrane. To study the effect of Onco-P20-induced fibroblast phenotypes on cancer cells simulating an in vivo situation, we used two different experimental systems: a fibroblast pre-treated conditioned medium (CM) and the trans-well permeable support (TW) for growth tumor and stromal cells on two different, but connecting, monolayers. Taking into consideration that previous studies evidenced that mesenchymal cells are capable of releasing into the microenvironment functional active paclitaxel for 24 h [41,42], in both cases, we replaced the Onco-P20-containing medium with fresh starved medium 48 h before collecting CM or starting the co-culture experiments. In both cases, due to the absence of paclitaxel and serum, cell proliferation depended exclusively on autocrine and paracrine activity of both cell populations. At the experimental end point (72 h), both systems clearly demonstrated that the carcinoma cells dose-dependently slowed their proliferation rate in the presence of Onco-P20-pretreated fibroblasts or their CM (Figure 7a,b). The anti-proliferative effects observed depended on the dose of the drug used to treat NHFs and on the relative fibroblast–carcinoma ratio, as demonstrated by the stronger reduction in A431 cell growth in the trans-well’s upper (smaller) compartment compared to the lower one. Furthermore, the co-culture data were confirmed using a patient-derived model assembled with NAF, CAF, and carcinoma cells from the same donor (Figure 7c) or NAF, CAF, and A431 cell lines for cases presenting with the unsuccessful isolation of tumor cells in vitro (Figure 7d). Overall, the data demonstrated that the sensitivity of CAFS to Onco-P20 was similar to that of normal fibroblasts.

## 4. Discussion

Skin diseases represent a significant health burden. Although epithelial skin cancers rarely metastasize, they grow invasively, and their removal often leads to severe functional and aesthetic impairments [62]. Therefore, there is a strong need for the development of cost-effective and well-tolerated therapies. The use of topical pharmacologic interventions aims to treat superficial forms of NMSC, decreasing the rate of disease progression and offering an effective nonsurgical prospective. An increasing number of studies have demonstrated that in tumors, the stroma is not simply a collection of enclosing cells without malignant function [9,63]. CAFs have been indicated as a key component of the cross-talk between cancer cells and their microenvironment. A dichotomous role has been attributed to CAFs. It is generally accepted that CAFs stimulate cancer cell growth and metastatic behavior [20,64]. Specifically, cancer cells utilize the CAF-secreted growth factors to facilitate their own survival and proliferation. However, the depletion of CAFs leads to invasive, undifferentiated tumors with enhanced hypoxia and cancer stem cells with reduced animal survival [20,21]. Thus, based on the idea that fibroblasts have an exceptional phenotypic plasticity, including the secretory phenotype, it has been proposed that CAFs could be re-oriented to an anti-tumor phenotype for therapeutic purposes. However, to efficiently direct CAF activity against tumors, we need to promote exclusively antineoplastic properties. Recent studies have demonstrated that CAFs are important modulators of the anti-tumor immune response [65,66]. Consequently, among the potential approaches, targeting the regulation of fibroblast–immune cell cross-talk represents an emerging strategy. Here, we demonstrated that a paclitaxel–hyaluronan conjugate could target cancer cells and surrounding fibroblasts, creating a synergism between direct and indirect anti-cancer effects. Previous studies have demonstrated that the Onco-P20 conjugate interacts with CD44, enters cells through a receptor-mediated mechanism, and exerts a concentration-dependent inhibitory effect on tumor cells [42,67,68]. In preclinical studies, HA–taxol has demonstrated reduced toxicity, enhanced tumor accumulation, and greater anti-tumor efficacy compared to free taxol in ovarian and bladder carcinomas [67,69]. However, in line with previous studies, we observed that during the carcinogenic process, keratinocytes did not significantly increase the level of CD44 on the membrane, and, consequently, Onco-P20 failed to demonstrate any significant advantage in the pharmacological activity compared to free paclitaxel in this cell type. Thus, we postulated that the modestly augmented receptor-mediated uptake is balanced by the smaller size and lipophilic nature of free paclitaxel, which easily diffuses across cell membranes in vitro [70,71,72]. Despite the absence of any advantage in receptor-mediated uptake, the use of a hydrophilic HA backbone is still of interest in vivo to overcome the limited aqueous solubility of paclitaxel. Independently of the kinetics of drug internalization, tumor cells under tubulin polymerization/depolymerization anti-cancer agent treatment undergo apoptotic cell death, whereas normal cells resist adopting a non-proliferative state [73,74]. The presence of functional p53 plays a crucial role in the level of cytotoxicity by paclitaxel. Cells lacking wild-type p53 respond through a p53-independent apoptosis, whereas cells expressing active p53 resist paclitaxel toxicity by growth arrest [75,76]. We consistently observed divergent outcomes when comparing normal and transformed keratinocytes. NHKs resist paclitaxel exposure, increasing G2/M arrest. In cells of mesenchymal origin, the switch to a quiescent state following paclitaxel exposure has been already described [77]. However, studies have mainly focused on mesenchymal stem cells and on the possibility of using paclitaxel-resistant cells as a reservoir of an active compound (as a “Trojan horse”) for local–regional drug delivery [78]. Very recently, Coccè and co-workers demonstrated that gingival mesenchymal stem cells treated with paclitaxel release vesicles containing an anti-cancer secretome in addition to free paclitaxel [79].

Here, we demonstrated that Onco-P20-treated dermal fibroblasts present a consistent reorganization of several intracellular signaling pathways. Most of the detected markers were related to the blocking of cell cycle progression, leading to a stress-induced non-proliferating status resembling a senescent-like phenotype. The observed G2 phase accumulation is a reversible state that, at least in vitro, does not necessarily fully correspond to senescence, since after taxol wash-out, cells resumed proliferation. Accordingly, we did not observe increases in senescence-associated β-galactosidase activity or parallel accumulation of p16 (data not shown), two markers stable of cell senescence. The lack of p16 is consistent with incomplete realization of the senescent program, since p21 over-expression is sufficient for senescent cell cycle arrest, and it is replaced by p16 after stable senescence is achieved [80]. The transitory nature of the Onco-P20-imposed phenotype could be explained by the fact that the DNA damage induced by paclitaxel could be repaired after drug removal [81]. This is an important point in consideration of the impact of treatment on tissue integrity and its long-term persistence. In fact, rapidly replaced cells, such as keratinocytes, are naturally protected from the accumulation of dysfunctional cells due to normal turnover by newly differentiating cells, whereas dermal fibroblasts are long-lived elements capable of proliferation under appropriate stimuli [82,83].

The presence of senescent cells in premalignant lesions in various mouse tumor models and human patients [84,85] is consistent with cellular senescence acting as a brake to the development of cancer. Hence, tumor cells must bypass the mechanisms that require a cellular senescence response in order to proliferate [16,86]. Senescence-associated phenotypes sensitize surrounding cells to senesce. Thus, it is possible that cancer cell senescence, the first line of cancer protection, rapidly spreads out to adjacent stromal cells. Accordingly, fibroblast senescence is a very early event in the carcinogenic process [87]. Whilst senescence acts as a tumor suppressor when activated in the epithelial compartment, the senescence of fibroblasts is frequently referred to as characteristic of CAFs, suggesting that the associated secretome could play a role in cancer progression. In apparent contradiction to the concept that fibroblasts in the tumor contest resemble senescent fibroblasts, CAF has been reported as a hyper-activated state associated with an augmented proliferation rate [88,89,90]. Moreover, CAFs are considered to be similar to myofibroblasts activated after injury, a situation that promotes mesenchymal cell expansion rather than proliferative arrest, as in the case of Onco-P20-treated cells. In addition, reprogramming activated fibroblasts or CAFs back into their dormant state has been proposed as a strategy for inhibiting tumorigenesis [16]. This implies converting CAFs back into a quiescent state similar to dermal fibroblasts in vivo. Thus, even if Onco-P20-treated fibroblasts displayed some biomarkers of senescence, the observed changes in gene expression did not fully overlap with those of the senescent phenotype of CAFs. One of the detrimental aspects of CAFs is the over-production and secretion of growth factors, which can induce proliferative signals within cancer cells. By contrast, dermal fibroblasts under continuous treatment with Onco-P20 decreased the production of some growth factors. Notably, we observed down-regulation of HGF and a simultaneous increase in most of the IGFBPs, which suggests a reduced bioavailability of IGF. Both HGF and IGF stimulate directly and indirectly (supporting angiogenesis) tumor growth [83,91]. HGF facilitates transformed epithelial cell migration and protects cancer cells from chemotherapeutic agents [83,92,93]. Moreover, IGF1 and IGF2 mediate cancer cells’ resistance to paclitaxel in murine models [94], suggesting that lowered IGF bioavailability could heighten chemotherapy. Interestingly, IGF1 diminishes in senescent and physiologically aged dermal fibroblasts [95,96], whereas its production is augmented in CAFs [97], confirming that CAFs are not simply senescent fibroblasts. Additionally, KGF, whose over-expression by fibroblasts enhances the proliferation of basal keratinocytes and suppresses epidermal cells’ terminal differentiation [98,99,100], decreased in the presence of Onco-P20. A minor production of growth factors may represent an autocrine control of proliferation when cell cycle progression is not possible, a situation that, in the context of carcinogenesis, could play an important role in paracrine regulation of tumor growth. Reduced mitogenic activity of fibroblasts could also counteract the increased number of CAFs in the reactive stroma. This idea is supported by a very recent study reporting fewer stromal CAFs in pancreatic cancer patients after treatment with nanoparticles containing paclitaxel [101]. Wnt signaling deregulation in human SCCs has been evidenced not only in cancer cells, but also in stromal fibroblasts [102], suggesting that the Wnt pathway might act in a paracrine fashion to promote skin carcinogenesis. Fibroblasts treated with Onco-P20 showed reduced β-catenin expression and increased production of soluble factors capable of diffusing similar Wnt pathway modulation to neighboring cells.

In line with the idea that when the cell cycle is blocked downstream, the pro-inflammatory pathway is over-activated [103], the most prominent feature of Onco-P20 growth-arrested fibroblasts was a consistent pro-inflammatory secretory profile. The pro-inflammatory gene signature has been evidenced in early hyperplastic lesions as well as in end-stage squamous cell carcinomas [31]. Immunomodulatory cytokines secreted by fibroblasts have a double-edged sword effect in cancer biology. Some cytokines over-expressed in Onco-P20-treated fibroblasts influence the mobilization of the local immune system, playing a relevant role in defense against cancer [104,105]. For example, CCL11 has been implicated in eosinophil recruitment and tissue necrosis [106]. IL1, IL6, and IL8 have a bimodal and dynamic behavior. Fibroblasts in healthy tissue secrete basal levels of IL6 and IL8 that act to maintain tissue homeostasis [107]. IL6 participates in the recruitment and polarization of macrophages, natural killer cells, and T cells, promoting immune control of cancer cells [6,108]. By contrast, high levels of IL6 and IL8 favor macrophage immunosuppressive phenotypes [109]. The augmented expression of IL6 in the peritumoral skin seems to be related to chronic UV exposure rather than to the presence of neoplastic cells, since IL6 is almost absent within BCC [22]. IL-1α in combination with the EGFR inhibitor can induce a T cell-dependent anti-tumor immune response in head and neck squamous cell carcinomas [110]. Acute inflammation, mainly the extensive production of IL1β by CAFs, significantly contributes to the efficacy of photodynamic therapy in cutaneous SCC [111]. Thus, inflammation may not always be “adverse” in the context of cancer, and the balance of individual members of the cytokine network can also contribute to the therapeutic response.

CAFs influence another major stromal component within tumors by directing tumor-associated macrophage (TAMs) polarization (M1, type I anti-tumor or M2, type II immunosuppressive phenotype) [112]. Even if this study does not evidence any specific predictive effect on macrophage polarization, an interesting patient-based study demonstrated that the antitumor effect of paclitaxel may occur in part via the reactivation of the immune response against cancer, guiding tumor-associated macrophages towards the M1-like anti-tumor phenotype [113]. In line with our study, the effect on TAM indicates that paclitaxel-based anti-tumor therapy does not target only cancer cells, but also heterotypic interactions. Moreover, in line with our data, strong IL1β activity occurs also in Onco-P20-treated macrophages. The advantage is that targeting tumor cells and normal stromal cells with the same molecule could offer a more effective and durable response, since normal cells appear to have a relatively stable genetic constitution in contrast to the genetically instable genomes of cancer cells. A controversial point is the transitory and reversible effect of Onco-P20 on fibroblasts. Although it is desirable to restore normal skin homeostasis at the end of treatment, there is still the possibility of the fibroblasts recovering the pro-tumor phenotype. On the other hand, the contemporary cytotoxic effect of taxol on the tumor should eliminate most cancer cells, significantly reducing the neoplastic cell-driven stimulation of fibroblasts.

## 5. Conclusions

Combined therapies, including molecules directed against stromal components, have been considered as a more efficient way to treat cancer. Overall, our experiments clearly demonstrated that CAF–cancer cell interactions, at least those mediated by secreted factors, could be re-directed by Onco-P20 into a tumor-suppressive function. The enhanced Onco-P20 uptake by fibroblasts compared to standard free paclitaxel also encourages the use of lower active therapeutic doses, thereby limiting the toxic effect on uninvolved cells.

## Figures and Tables

**Table 1:** Gene expression analysis of growth factors and CAF markers in Onco-P20-treated fibroblasts after 2 weeks of continuous treatment. Data represent x-fold increase or decrease with respect to untreated cells. Results are mean ± SD of seven different experiments performed with fibroblasts isolated from independent donors. * *p* < 0.05 and ** *p* < 0.001.

Target mRNA	Onco-P20
	(x-Fold Change)
HGF	0.44 ± 0.36 **
IGF	0.72 ± 0.97
bFGF	1.21 ± 0.51
KGF	1.41 ± 1.82
EGF	0.66 ± 0.53
VEGF	0.69 ± 0.29
IGFBP3	2.06 ± 2.05 **
IGFBP4	2.48 ± 0.59 *
IGFBP5	2.14 ± 1.9 *
IGFBP6	2.10 ± 0.54 *
IGFBP7	1.62 ± 0.68 *
Wnt5a	2.48 ± 2.77 *
DKK1	4.22 ± 2.38 **
SFRP2	7.60 ± 7.51 *
TGFb	0.87 ± 0.24
PDGFα	0.69 ± 0.71
PDGFb	0.57 ± 0.25 **
αSMA	1.14 ± 0.66
FAP1	4.06 ± 5.67 *
IL1α	18.6 ± 15.9 **
IL1b	34.6 ± 43.8 **
IL6	5.84± 7.20 *
IL8	109.2 ± 105.0 *

**Table 2:** Results of human inflammation genes array after 2 weeks of fibroblast treatment with Onco-P20 (or not control cells). Results are the mean ± SD of seven different experiments performed with fibroblasts isolated from independent donors. * *p* < 0.05 and ** *p* < 0.001. Not detected.

Target mRNA	Ctrl	Onco-P20
A2M	2.16 ± 4.53	12.6 ± 9.97
ADRB1	ND	ND
ADRB2	1.78 ± 3.07	3.87 ± 7.09 **
ALOX12	ND	ND
ALOX5	ND	ND
ANXA1	1.70 ± 0.33	3.33 ± 1.06 **
ANXA3	3.56 ± 2.07	3.40 ± 2.08
ANXA5	1.56 ± 0.31	2.02 ± 0.54
B2M	0.43 ± 0.70	1.89 ± 1.21 **
BDKRB1	0.23 ± 0.12	7.65±6.20 *
BDKRB2	0.33 ± 6.20	6.06 ± 5.31 *
CACN1C	1.4 ± 0.69	1.7 ± 1.48
CACN2D1	ND	ND
CACNB2	1.07 ± 2.01	2.04 ± 1.63
CACNB4	1.63 ± 2.95	4.07 ± 6.04
CASP1	1.72 ± 0.65	4.84 ± 0.60
CD40	2.57 ± 1.72	9.96 ± 4.45 **
CD40LG	ND	ND
CES1	0.27 ± 0,19	0.89 ± 0.9
CYSLTR1	ND	1.88 ± 2.01
HPDG	ND	ND
HRH1	2.91 ± 0.99	2.33 ± 1.13
HRH2	ND	ND
HRH3	ND	ND
HTR3A	ND	ND
HTR3B	ND	ND
ICAM1	0.42 ± 0.25	9.57 ± 6.17 **
IL13	ND	ND
IL1R1	0.98 ± 0.66	3.87 ± 2.12 *
IL1R2	ND	ND
IL1RAPL2	ND	ND
IL1RL1	ND	ND
IL2RA	ND	ND
IL2RB	ND	ND
IL2RG	ND	ND
ITGAL	ND	ND
ITGAM	ND	ND
ITGB1	1.68 ± 0.29	2.96 ± 1.5
ITGB2	0.51 ± 0.32	2.7 ± 1.74 *
KLK1	ND	ND
KLK14	0.55 ± 0.93	6.08 ± 2.31 **
KLK15	ND	ND
KLK2	ND	ND
KLK3	ND	ND
KLKB1	ND	ND
KNG1	ND	ND
LTA4H	1.21 ± 0.25	2.34 ± 0.78 *
LTB4R	2.15 ± 0.87	2.82 ± 1.68
LT4R2	0.81 ± 0.63	1.94 ± 1.55
LTC4S	0.53 ± 0.37	0.53 ± 0.28
MAPK1	0.63 ± 0.48	0.62 ± 0.38
MAPK14	1.11 ± 0.51	1.95 ± 1.01
MAPK3	0.96 ± 0.27	1.40 ± 0.47
MAPK8	1.33 ± 0.71	2.49 ± 0.99
MC2R	ND	ND
NFKB1	0.28 ± 0.15	0.60 ± 0.38
NOS2A	ND	ND
NR3C1	1.3 ± 0.33	1.9 ± 0.86
PDE4A	0.83 ± 0.24	1.03 ± 0.21
PDE4B	0.29 ± 0.13	2.59 ± 2.75
PDE4C	ND	ND
PDE4D	0.33 ± 0.42	4.50 ± 4.30
PLA2G10	ND	ND
PLA2G1B	ND	ND
PLA2G2A	ND	ND
PLA2G2B	ND	ND
PLA2G2D	ND	ND
PLA2G4C	0.19 ± 0.15	2.63 ± 1.94 *
PLA2G5	ND	ND
PLA2G7	ND	ND
PDE4D	0.33 ± 0.43	4.50 ± 4.3
PLCB2	ND	ND
PLCB3	0.77 ± 0.21	0.66 ± 0.15
PLCB4	2.01 ± 1.77	1.23 ± 0.79
PLCD1	1.19 ± 0.54	1.69 ± 0.47
PLCE1	0.60 ± 0.63	0.36 ± 0.35
PLCG1	7.26 ± 2.47	13.9 ± 1.18
PLCG2	1.90 ± 1.28	2.99 ± 2.04
PTAFR	0.15 ± 0.34	1.08 ± 1.52
PTGDR	0.09 ± 0.2	8.30 ± 6.32 *
PTGER2	0.60 ± 0.41	4.06 ± 4.27
PTGER3	2.5 ± 2.65	5.2 ± 2.97
PTGFR	1.0 ± 0.41	3.3 ± 2.43
PTGIR	0.80 ± 0.47	1.55 ± 0.54 *
PTGIS	0.83 ± 0.84	2.57 ± 2.47
PTGS1	0.65 ± 0.74	1.1 ± 0.50
PTGS2	0.42 ± 0.49	6.42 ± 6.88
TBXA2R	0.8 ± 0.27	1.7 ± 0.78 *
TBXAS1	ND	ND
TNF	ND	ND
TNFRSF1A	1.4 ± 0.52	1.9 ± 0.93
TNFRSF1B	0.67 ± 0.54	2.48 ± 1.07 **
TNFRSF13B	1.2 ± 1.42	12.8 ± 9.62 *
VCAM1	0.3 ± 0.53	13.2 ± 21.6

**Table 3:** Semi-quantitative analysis of multiple cytokines secreted by Onco-P20-treated fibroblasts after 2 weeks of continuous treatment. Data represent x-fold increase or decrease with respect to untreated cells. Results are the mean ± SD of four different experiments performed with fibroblasts isolated from independent donors. * *p* < 0.05 and ** *p* < 0.001.

Target mRNA	Onco-P20
	(x-Fold Change)
CCL11	2.65 ± 1.0 *
CCL24	1.16 ± 0.87
GCSF	0.57 ± 0.34
GMCSF	1.03 ± 0.44
INGγ	1.44 ± 0.61
IL1α	1.55 ± 1.09
IL1β	2.11 ± 1.58
IL2	1.35 ± 0.60
IL3	1.03 ± 0.59
IL4	2.37 ± 2.12
IL6	8.65 ± 4.84 *
IL7	0.85 ± 0.61
IL8	4.40 ± 1.64 **
IL10	1.22 ± 0.61
IL11	0.90 ± 0.43
IL12p40	0.96 ± 0.71
IL12p70	2.19 ± 2.20
IL13	0.73 ± 0.57
CCL1	0.26 ± 0.31 *
TIMP-2	0.81 ± 0.2

## Data Availability

The datasets used or analyzed during the current study are available from the corresponding author on reasonable request.

## Supplementary Materials

The following are available online at https://www.mdpi.com/article/10.3390/biomedicines9060597/s1.
