# Supplementary figures and images for: Simultaneous Targeting Tumor Cells and Cancer-Associated Fibroblasts with a Paclitaxel–Hyaluronan Bioconjugate: In Vitro Evaluation in Non-Melanoma Skin Cancer
Source: Biomedicines. 2021 May 24;9(6):597. doi: 10.3390/biomedicines9060597 (PMC8225214; doi:10.3390/biomedicines9060597)

Fig. S1

a

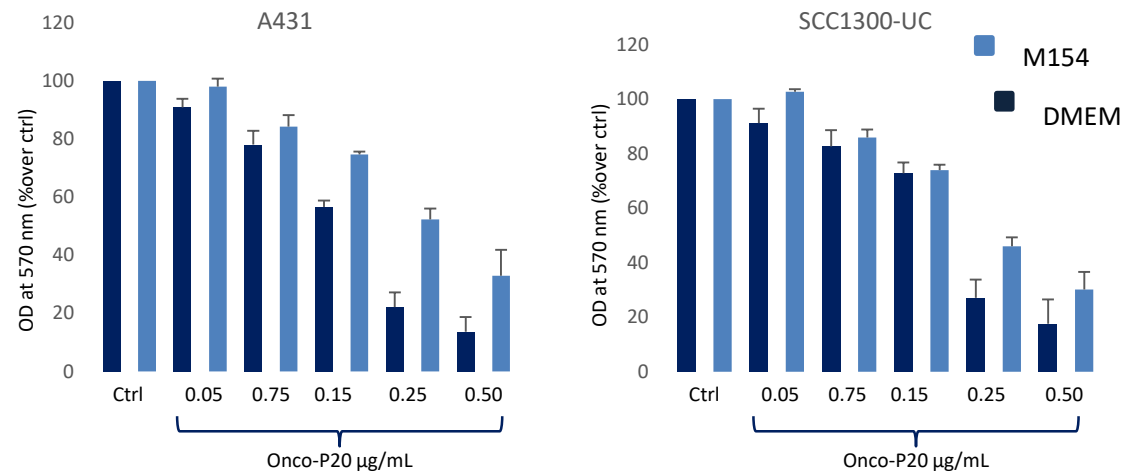

b

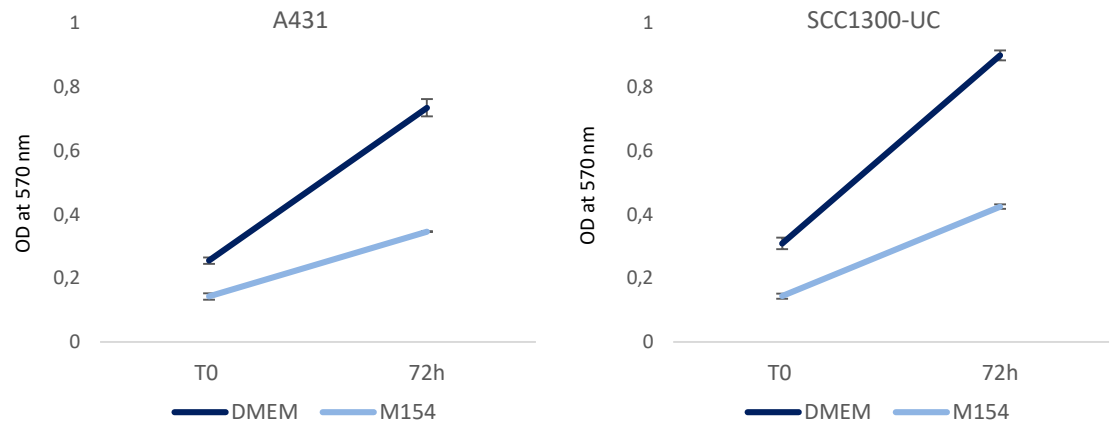

Fig. S2

A431

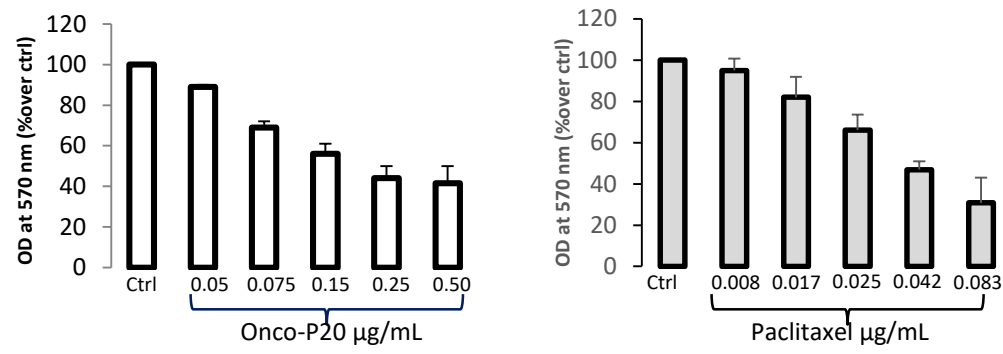

SCC1300-UC

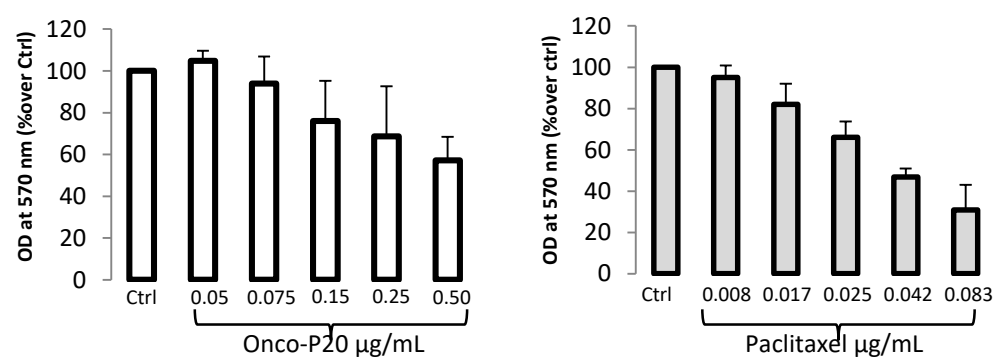

NHK

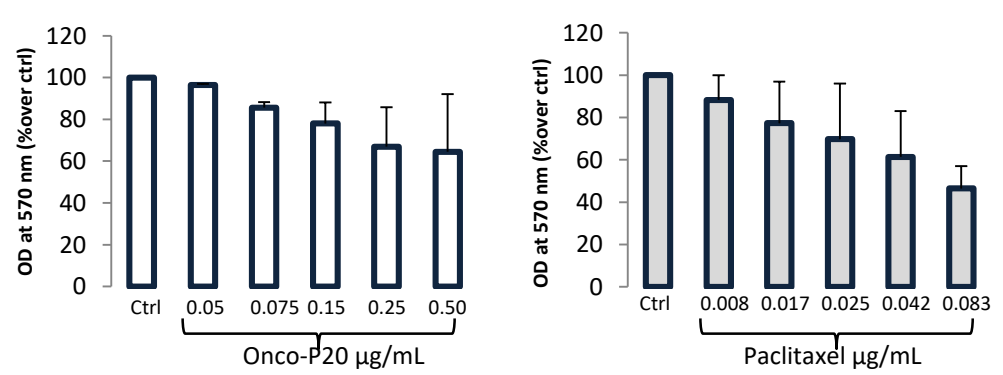

Fig. S3

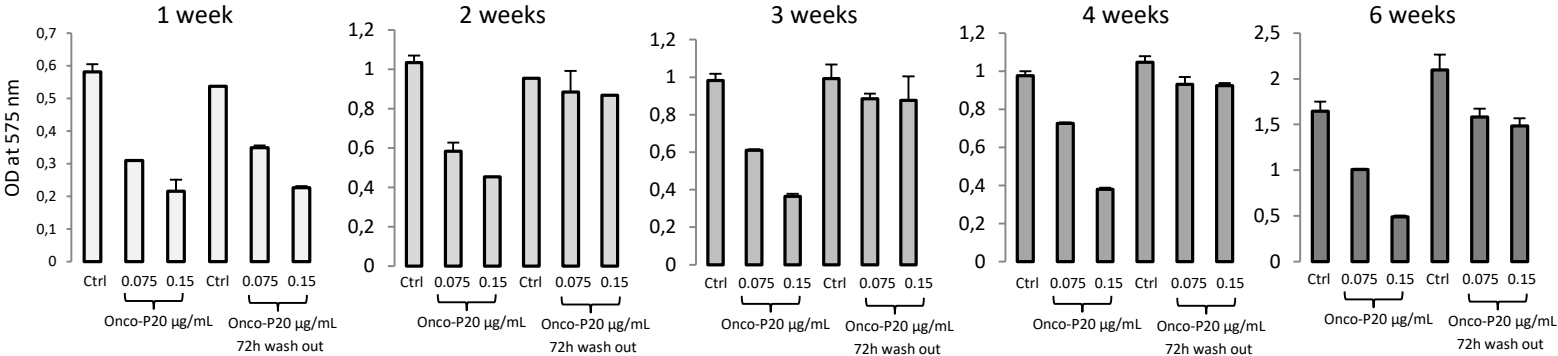

Fig. S4

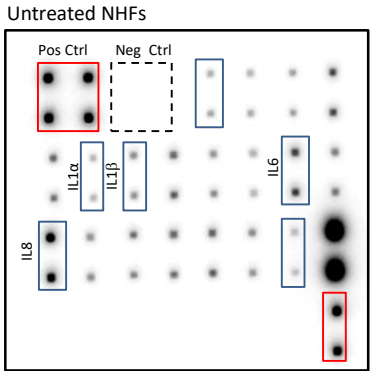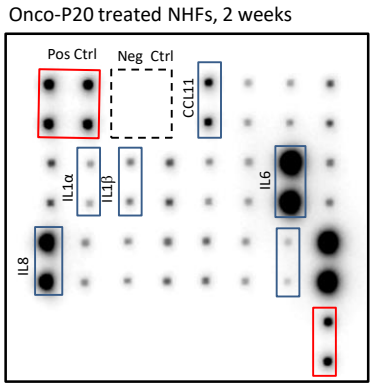

Supplement: Supplementary file 1 [file biomedicines-09-00597-s001.zip › biomedicines-1189205-supplementary.pdf]
